# Risk of suicide and self-harm in university students entering different university programs – a national register-based cohort study in Sweden

**DOI:** 10.1007/s00127-023-02484-2

**Published:** 2023-05-07

**Authors:** Christine Takami Lageborn, Johan Bjureberg, Jie Song, Bo Runeson, Jette Möller, Rickard Ljung, Marie Dahlin

**Affiliations:** 1 Centre for Psychiatry Research, Department of Clinical Neuroscience, Karolinska Institutet and Stockholm Health Care Services, Region Stockholm, Norra Stockholms Psykiatri, Vårdvägen 1, 112 81 Stockholm, Sweden; 2 Department of Medical Epidemiology and Biostatistics, Karolinska Institutet, Stockholm, Sweden; 3 Centre for Psychiatry Research, Karolinska Institutet and Stockholm Health Care Services, Stockholm County Council, Stockholm, Sweden; 4 Department of Psychology, Stanford University, Stanford, CA USA; 5 Department of Global Public Health, Karolinska Institutet, Stockholm, Sweden; 6 Institute of Environmental Medicine, Unit of Epidemiology, Karolinska Institutet, Stockholm, Sweden

**Keywords:** Suicide, Self-harm, University, Student, Program

## Abstract

**Purpose:**

It is not known whether the elevated suicide risk in certain occupations, such as health care professionals, is partly attributable to a selection of individuals with prior vulnerability. We aimed to determine the risk of suicide and self-harm already in students entering different university programs.

**Methods:**

We used national registers to identify 621,218 Swedish residents aged 18–39 years with registration for a university program 1993–2013. Outcomes were suicide and self-harm within three years. We applied logistic regression analyses to calculate odds ratios (OR) and 95% confidence intervals (CI) of risk of suicide and self-harm, with the Education program category as a reference. Results were adjusted for sex, age, time period and for history of hospitalization due to mental disorder or self-harm, as a measure of previous vulnerability. In the second step, we stratified results by sex.

**Results:**

There was a higher risk of suicide (OR 2.4) in female nursing students and natural science students (OR 4.2) and of self-harm in female and male Nursing/Health care students (OR range 1.2 –1.7). Subcategorization into nursing students only strengthened the association with self-harm for both sexes. Prior vulnerability did not fully explain the increased risk.

**Conclusion:**

The elevated risk of suicide in nursing and health care occupations partly has its onset in vulnerability factors present before or emerging during university studies. Increased efforts in identifying and treating mental disorders and preventing self-harm in university students could be an important step in preventing future suicides.

## Introduction

In working professionals within occupations requiring a university degree an increased risk of suicide has repeatedly been reported in the medical and health-related field [1–4]. Several explanations have been proposed, such as accessibility to means of suicide and a reluctance to seek help for mental health problems [2, 5, 6]. However, it is not known whether the elevated risk may be influenced by a selection of vulnerable individuals choosing that career path.

Nurses and physicians, especially female physicians, have been observed to have elevated risks of suicide [2, 4, 7]. A U.S. study on suicide risk in nurses and physicians found a higher suicide risk in female nurses compared with the general population, whereas no increased suicide risk was seen in physicians of either gender [8]. Studies conducted also on other occupational groups have shown elevated suicide risks in veterinarians, pharmacists, dentists and persons working in arts or media [9, 10].

Established main risk factors for suicide include previous attempts, a family history of suicide and having a mental disorder at the time [11]. Low educational attainment is also associated with a higher risk of suicide, whereas high educational attainment has been associated with a lower risk, at least in men [12, 13].

According to the stress-diathesis model, suicidal behavior occurs due to an interaction between a predisposing vulnerability and life stressors [14]. It is possible that persons with a higher baseline vulnerability are more likely to choose certain educational paths. For instance, a previous study showed that individuals with bipolar disorder or siblings of individuals with bipolar disorder or schizophrenia are more highly represented in creative professions [15].

University programs aimed at future healthcare occupations involve varying degrees of practical training in hospitals and may expose students to meetings with ill or dying patients. They may thus already during training face stressors similar to those encountered by working healthcare professionals. High levels of self-reported depressive symptoms have been described in university students in health-oriented programs, veterinary students, and students of law, social sciences and engineering [16–20].

While the suicide rate is highest among those with a lower educational level, a Swedish national register-based cohort study from our group found that the suicide risk is higher during ongoing studies than when having achieved a university education [21]. Yet, there is a scarcity of studies investigating whether suicide risk varies for different university programs. A Japanese study, based on survey data of suicide decedents collected over 23 years from multiple universities, reported a higher suicide risk in medical students and a lower risk in students majoring in humanities [22].

In this nationwide cohort study, we aimed to determine the risk of suicide and self-harm in university students within a three-year period of entering different university programs. We examined men and women separately and took previous hospitalizations for mental disorder or self-harm into account to evaluate the influence of vulnerability prior to the study start.

## Method

### Participants

The study population consisted of 621,218 persons (55.8% women) aged 18–39 years (mean age at registration was 22.6 years; SD = 4.6) who registered for a university program during 1993–2013 in Sweden. Free-standing university courses, master-level programs, other postgraduate programs and PhD studies were not included.

We used data from multiple Swedish national registers by linkage through the unique identification number assigned to all residents in Sweden. Firstly, the Total Population Register was used to retrieve data on eligible individuals, date of birth and sex [23]. Data on university program and time periods of being a student was retrieved from The Administrative data for University and Higher Education (AUH), held by Statistics Sweden. Suicide data was retrieved from the National Cause of Death Register and records on inpatient care for self-harm and mental disorders were retrieved from the National Patient Register (NPR). These are both held by the Swedish National Board of Health and Welfare. All data was pseudonymized and the researchers had no information on the identity of individuals.

### Exposure

There is no gold standard for how to group university programs into broader categories. We adopted AUH’s categorization of university programs with a minimum duration of three years: Theology/Humanities, Law/Social sciences, Education, Natural sciences, Technology, Agriculture/Forestry, Medicine/Dentistry, Nursing/Health care, Art studies or Miscellaneous (See Table 1 for a list of included programs). Alternative categorizations were considered, but not judged to be superior. All students were followed for three years following registration at a university program.

**Table 1:** Subjects included in the university programs in the broader program categories, followed by the two subgroup programs, and program length

University program category	Included programs and normal course duration
Theology/Humanities	3 years: Theology, ancient cultures, linguistics, Arabic, archeology, Aramaic/Syrian, archival science, Bulgarian, library and information science, Bosnian/Croatian/Serbian, Danish, dance and theater science, English, Estonian, aesthetics, ethnology, Finnish, philosophy, multilingual subjects, French, film science, Greek, Hebrew, other historic-philosophic subjects, Hindi, history, history of ideas, Indonesian, Indology and Sanskrit, Italian, Japanese, communication and informatics, Journalism, Chinese, Korean, Kurdish, conservation, art history, culture science, Latin, Latvian, Lithuanian, literary science, media and communication science, media production, music science, Dutch, Greek, Polish, Persian, Portuguese, rhetoric, Romanian, Religion, Russian, Spanish, other languages, Swedish as a second language, Swedish/Nordic languages, Swahili, Tamil, Sign language, Thai, Tibetan, Czech, theology, translation and interpretation, German, Hungarian
Law/Social sciences	3 years: Administration and management, behavioral science, economic history, economy/administration, business economics, Peace and conflict studies, science of the disabled, informatics, criminology, culture and societal geography, studies of countries, leadership and organization skills, domestic science, human rights, political economy, science of education, social sciences, sociology, social anthropology, social welfare work, statistics, political science, student counselling, tourism, general science of education/didactics/theoretical/practical aesthetical subjects 4.5 years: Law and jurisprudence 5 years: Psychology program
Education	3 years: Student counselor program At least 3.5 years: Teacher education programs (pre-school, primary school, high school)
Natural sciences	3 years: Bachelor program in pharmacy, biology, biotechnology, pharmacy science, pharmacology, fishing and water supply, physics, earth science and natural geography, pet science, chemistry, grocery science, agriculture, mathematics, medical biology, mathematical statistics, environmental science, miscellaneous within the natural sciences, nutrition, forestry, garden science
Technology	3 years: Applied technology program, mate degree program, nautical science, mechanical engineer program, sea captain program, college engineer program, fire engineer program, architectural program, architecture, automation technology, rock and mineral technology, science of construction, computer technology, electronics, energy technology, electrotechnology, physical planning, vessel technology, geographical information technology and cadastral survey, industrial economy/organization, chemical technology, landscape architecture, science of materials, machine technology, space science, technology of societal construction, miscellaneous subjects within technology, engineering physics, tree physics and tree technology, textile technology, water and water supply construction
Agriculture/Forestry	3 years: Gardening engineer program, forest technician program, forester program, landscape engineer program, horticulturist program, hippologist program, animal paramedic degree program, agronomist degree program
Medicine/Dentistry	At least 3 years: Study program in medicine (5.5 years), study program in dentistry, optician program, veterinary school, Biomedical lab science, animal nursing, medicine, miscellaneous in medicine, medical technology, dentistry techniques and oral health
Nursing/Health care	3 years: Dental hygienist program, dental technician program, social care program, nursing school, physiotherapist program, medical physics program, radiographer program, prosthetist program, speech therapist program, dietician program, biomedical scientist program, midwife program, audiologist program, occupational therapist program, physiotherapist program, public health science, the development of health care, nursing/science of nursing, miscellaneous in nursing, therapy, rehabilitation and dietary treatment
Arts studies	3 years: Organist program, stage, setting and media, music, arts and design, dance program, circus, dance and design, authorship (composition), free arts, film, handicraft, choreography, musical drama and staging and figuration, music, direction, stage and media, theater, film, and dance
Miscellaneous	3 years: Polytechnic degree program, officer program, general bachelor program, human work science and ergonomics, children and youth studies, health care, gender studies, physical education, science of war, shipping/navigation, miscellaneous in the field of transportation, technology from a societal perspective, miscellaneous multidisciplinary studies, exchange studies
Subgroup programs	
Medical students	5.5 years (6 years since 2021): Study program in medicine. After the university program, which includes clinical training, there is a mandatory 18 months internship to become a licensed physician
Nursing students	3 years: Study program in nursing. After three years a Degree of Bachelor is attained, and the student can immediately get a nursing license

To also investigate risk specifically amongst medical students and nursing students, we carried out an analysis restricted to students in these courses. They came from a subset of Medicine/Dentistry students and Nursing/Health care students. The study program in medicine in Sweden was, until year 2021, 5.5 years long and included clinical training. A student admitted to the study program in medicine can follow the program until the end with no need to re-apply, required that all course requirements are fulfilled. The study program in nursing is 3 years long and if the program is followed through it results in a Degree of Bachelor as well as a nursing license.

Students in the Education program category were used as a reference, consistent with two previous studies on suicide risk in different occupations [1, 2], which would facilitate comparison. The Miscellaneous category was omitted from the analyses since it consisted of many disparate and unknown programs.

### Outcomes

Suicide was defined as an underlying cause of death recorded in the National Cause of Death Register according to the International Statistical Classification of Diseases and Related Health Problems (ICD) as: (ICD 9: E950–E959; ICD-10: X60–X84) or unnatural death of undetermined intent (ICD9: E980-E989, ICD-10: Y10-Y34). Deaths of undetermined intent were included to avoid the risk of underestimation of suicides as a proportion of the deaths by suicide may have been misclassified [24].

Self-harm was defined as hospitalization due to a self-harm event recorded in the NPR as either of intentional (ICD-9: E950–E959, ICD-10: X60–X84) or of undetermined intent (ICD 9: E980–E989, ICD-10: Y10–Y34). The term self-harm instead of suicide attempt was used since suicidal intent cannot be known from registry data.

### Covariates

We adjusted for age and for the calendar year at the time of registration. For the total groups of students in different categories we also adjusted for sex. Vulnerability prior to entering a university program was defined as having a lifetime history of at least one hospitalization due to self-harm and/or due to mental disorder prior to the year of registration. Self-harm was defined as a diagnosis in the NPR as stated above. Mental disorder was defined as hospitalization due to any of the ICD-9 codes 295–319 or ICD-10 codes F10–F99.

## Statistical analysis

We used descriptive statistics to outline the distribution of population characteristics such as sex, age, and previous hospitalization for any mental disorder or self-harm. The associations between university program category and risk of suicide or first-time self-harm event were estimated by logistic regression analyses to yield odds ratios (ORs) with 95% confidence intervals (CI), interpreted as relative risk (RR) due to both outcomes being rare [25].

Firstly, crude and adjusted odds ratios for each outcome within three years of registration in a university program were estimated including age, sex and calendar year of entering university as covariates; and second a full model was estimated adjusting for age, sex, calendar year and previous hospitalization for mental disorder and self-harm. Similar crude, adjusted, and fully adjusted models were then run separately for men and women. Students in the Education program category constituted a reference group. Absolute risk differences were calculated for both suicide and self-harm for all university program categories and the subcategories of medical students and nursing students, stratified by sex and with 95% confidence intervals.

Lastly, a sub-analysis of nursing students and medical students was conducted with crude, partly and fully adjusted models. Data management and statistical analyses were performed with SAS version 9.4 (SAS Institute Inc. Cary, NC, USA) and Stata version 16.1 (StataCorp, College Station, TX, USA).

## Results

### Descriptive characteristics

Descriptive characteristics of the study population are presented in Table 2 for each university program category as total, followed by men and women separately. The Technology category held the largest number of students, 204,592, constituting one-third of all students. The lowest number of students, 7,425 (1.2%), was found in Agriculture/Forestry, not considering the Miscellaneous category with 7,228 students.

**Table 2:** Characteristics of students in different university program categories and subgroup programs who had a first university program registration between 1993 and 2013

University program category	Group	*N*	Mean age (SD)^a^	Hospitalization prior to university		Outcome within three years	
				Mental disorders *n* (%)	Self-harm *n* (%)	Suicide *n*	Self-harm *n* (%)
Education	Total	135,638	23.6 (5.2)	3,614 (2.7)	1,217 (0.9)	21	363 (0.3)
	Women	103,174 (76.1)	23.7 (5.3)	2,833 (2.7)	1,069 (1.0)	9	312 (0.3)
	Men	32,464 (23.9)	23.4 (4.6)	781 (2.4)	148 (0.5)	12	51 (0.2)
Theology/Humanities	Total	20,635	21.7 (3.4)	752 (3.6)	224 (1.1)	7	67 (0.3)
	Women	12,197 (59.1)	21.6 (3.4)	479 (3.9)	174 (1.4)	< 5	53 (0.4)
	Men	8,438 (40.9)	21.9 (3.4)	273 (3.2)	50 (0.6)	< 5	14 (0.2)
Law/Social sciences	Total	110,866	22.2 (4.2)	3,749 (3.4)	1,210 (1.1)	18	298 (0.3)
	Women	69,545 (62.7)	22.4 (4.2)	2,580 (3.7)	991 (1.4)	6	214 0.3)
	Men	41,321 (37.3)	22.0 (3.7)	1,169 (2.8)	219 (0.5)	12	84 (0.2)
Natural sciences	Total	17,649	21.9 (4.0)	427 (2.4)	128 (0.7)	< 5	45 (0.3)
	Women	10,799 (61.2)	22.1 (4.2)	254 (2.4)	89 (0.8)	< 5	32 (0.3)
	Men	6,850 (38.8)	21.6 (3.6)	173 (2.5)	39 (0.6)	< 5	13 (0.2)
Technology	Total	204,592	21.6 (3.8)	3,191 (1.6)	724 (0.4)	68	247 (0.1)
	Women	49,655 (24.3)	21.5 (3.9)	886 (1.8)	290 (0.6)	< 5	93 (0.2)
	Men	154,937 (75.3)	21.6 (3.7)	2,305 (1.5)	434 (0.3)	> 63	154 (0.0)
Agriculture/Forestry	Total	7,425	22.7 (3.8)	119 (1.6)	35 (0.5)	< 5	7 (0.1)
	Women	4,082 (55.0)	22.2 (3.9)	38 (0.9)	27 (0.7)	< 5	6 (0.1)
	Men	3,343 (45.0)	23.2 (3.7)	81 (2.4)	8 (0.2)	< 5	1 (0.0)
Medicine/Dentistry	Total	14,951	21.6 (3.4)	308 (2.1)	110 (0.7)	5	27 (0.2)
	Women	9,199 (61.5)	21.4 (3.2)	221 (2.4)	85 (0.9)	< 5	19 (0.2)
	Men	5,752 (38.5)	22.0 (3.7)	87 (1.5)	25 0.4)	< 5	8 (0.1)
Nursing/Health care	Total	93,417	24.6 (5.5)	3,318 (3.6)	1,308 (1.4)	22	331 (0.4)
	Women	79,885 (85.5)	24.6 (5.6)	2,921 (3.7)	1,200 (1.5)	17	295 (0.4)
	Men	13,532 (14.5)	24.7 (5.0)	397 (2.9)	108 (0.8)	5	36 (0.3)
Art studies	Total	8,817	23.0 (3.5)	244 (2.8)	72 (0.8)	< 5	22 (0.3)
	Women	4,538 (51.5)	22.9 (3.3)	140 (3.1)	49 (1.0)	< 5	12 (0.3)
	Men	4,279 (48.5)	23.1 (3.6)	104 (2.4)	23 (0.5)	< 5	10 (0.2)
Miscellaneous	Total	7,228	22.0 (3.5)	174 (2.4)	56 (0.8)	0	26 (0.4)
	Women	3,643 (50.4)	21.8 (3.6)	87 (2.4)	41 (1.1)	0	17 (0.5)
	Men	3,585 (49.6)	22.2 (3.5)	87 (2.4)	15 (0.4)	0	9 (0.3)
Subgroup programs							
Medical students	Total	8,984	21.4 (3.1)	186 (2.1)	75 (0.8)	< 5	17 (0.2)
	Men	3,968 (44.2)	21.8 (3.4)	55 (1.4)	19 (0.5)	< 5	7 (0.2)
	Women	5,016 (55.8)	21.2 (2.9)	131 (2.6)	56 (1.1)	< 5	10 (0.2)
Nursing students	Total	62,073	24.8 (5.6)	2,399 (3.9)	956 (1.5)	15	264 (0.4)
	Men	8,473 (13.7)	25.0 (5.0)	282 (3.3)	79 (0.9)	< 5	29 (0.3)
	Women	53,600 (86.4)	24.8 (5.7)	2,117 (3.9)	877 (1.6)	> 10	235 (0.4)
Total	All students	621,218 (100)	22.6 (4.6)	15,896 (2.6)	5,084 (0.8)	149	1,433 (0.2)
	Women	346,717 (55.8)	23.1 (5.0)	10,482 (3.0)	4,015 (1.2)	43	1,053 (0.3)
	Men	274,501 (49.2)	22.1 (4.0)	5,414 (2.0)	1,069 (0.4)	106	380 (0.1)

^a^Mean age (SD) at first registration in a university program

All categories except for Technology held a majority of female students. Nursing/Health care held the largest proportion of female students, 85.5%, whereas there was almost an equal gender distribution in Art studies and Agriculture/Forestry.

The prevalence of having at least one hospitalization for any mental disorder prior to university studies varied between categories (Table 2). The highest prevalence in the broader categories was in Theology/Humanities, and in Nursing/Health care, both 3.6%. The subcategory of nursing students had the highest prevalence of prior mental disorder, 3.9%. Technology students had the lowest prevalence of prior mental disorder, 1.6%. A similar pattern was seen regarding previous hospitalization due to self-harm where the highest prevalence was in Nursing/Health care, 1.4%, and in the subcategory of nursing students, 1.5%. Technology students had the lowest prevalence also of prior self-harm, 0.4%.

### Suicide after registration in different university programs

Within three years of entering a university program category 149 suicides were registered in total (Table 2). Men accounted for a majority of the suicides, *n* = 106 (71%). For exposure groups in which fewer than 5 suicides occurred the exact number was replaced by < 5.

Results adjusted for age and period and stratified by sex showed a higher risk of suicide in female students of Natural sciences, OR 4.5** (**95% CI 1.3–14.9), and in Nursing/Health care students, OR 2.4 **(**95% CI 1.1–5.3) (Table 3). After further adjustment for prior vulnerability, the suicide risk remained elevated compared to the reference category.

**Table 3:** Odds ratios (ORs) with 95% confidence intervals (CI) of suicide and self-harm for the total group, and for women and men separately within three years after registration in a university program category for those with a first registration during 1993–2013

University program category	Group	Suicide			Self-harm		
		Crude (95% CI)	Adjusted (95% CI)^a^	Adjusted for prior vulnerability (95% CI)^b^	Crude (95% CI)	Adjusted (95% CI)^a^	Adjusted for prior vulnerability (95% CI)^b^
Education (reference)	–	1	1	1	1	1	1
Theology/Humanities	Total	2.2 (0.9, 5.2)	1.9 (0.8, 4.5)	1.7 (0.7, 4.1)	1.2 (0.9, 1.7)	1.1 (0.9, 1.5)	1.0 (0.8, 1.4)
	Women	2.8 (0.8, 10.4)	3.0 (0.8, 11.6)	2.7 (0.7, 10.4)	1.4 (1.1, 1.9)	1.2 (0.9, 1.7)	1.2 (0.9, 1.6)
	Men	1.3 (0.4, 4.0)	1.3 (0.4, 4.2)	1.3 (0.4, 4.0)	1.1 (0.6, 1.9)	0.9 (0.5, 1.6)	0.8 (0.4, 1.5)
Law/Social sciences	Total	1.0 (0.6, 2.0)	0.9 (0.5, 1.8)	0.9 (0.5, 1.6)	1.0 (1.0, 1.2)	0.9 (0.8, 1.1)	0.9 (0.8, 1.0)
	Women	1.0 (0.5, 2.8)	1.0 (0.4, 3.0)	0.9 (0.3, 2.7)	1.0 (0.9, 1.2)	0.9 (0.8, 1.1)	0.9 (0.6, 1.3)
	Men	0.8 (0.4, 1.7)	0.8 (0.4, 1.9)	0.8 (0.3, 1.8)	1.3 (0.9, 1.8)	1.1 (0.8, 1.6)	1.0 (0.7, 1.5)
Natural sciences	Total	1.5 (0.5, 4.3)	1.3 (0.4, 3.8)	1.3 (0.8, 2.2)	1.0 (08, 1.3)	0.9 (0.7, 1.2)	0.9 (0.7, 1.3)
	Women	4.2 (1.3, 13.8)	4.5 (1.3, 14.9)	4.7 (1.4, 15.6)	1.0 (0.7, 1.4)	0.9 (0.6, 1.3)	0.9 (0.6, 1.3)
	Men	NA	NA	NA	1.2 (0.7, 2.2)	1.0 (0.5, 1.9)	1.0 (0.5, 1.8)
Technology	Total	2.1 (1.3, 3.5)	1.3 (0.8, 2.2)	1.3 (0,8, 2.2)	0.5 (0.4, 0.5)	0.6 (0.5, 0.7)	0.6 (0.5, 0.7)
	Women	0.5 (0.1, 2.1)	0.5 (0.1, 2.3)	0.5 (0.1, 2.5)	0.6 (0.5, 0.8)	0.6 (0.5, 0.7)	0.6 (0.5, 0.8)
	Men	1.2 (0.6, 2.1)	1.2 (0.6, 2.2)	1.2 (0.7, 2.3)	0.6 (0.5, 0.9)	0.7 (0.5, 1.0)	0.7 (0.5, 1.0)
Agriculture/Forestry	Total	0.9 (0.1, 6.5)	0.7 (0.1, 5.0)	0.7 (0.1, 5.4)	0.4 (0.2, 0.8)	0.4 (0.2, 0.8)	0.4 (0.2, 0.9)
	Women	NA	NA	NA	0.5 (0.2, 1.1)	0.5 (0.2, 1.0)	0.5 (0.2, 1.1)
	Men	0.8 (0.1, 6.2)	0.8 (0.1, 6.3)	0.9 (0.1, 6.8)	0.2 (0.3, 1.4)	0.2 (0.0, 1.4)	0.2 (0.0, 1.6)
Medicine/Dentistry	Total	2.2 (0.8, 5.7)	1.9 (0.7, 5.0)	1.9 (0.7, 5.0)	0.7 (0.5, 1.0)	0.7 (0.5, 1.0)	0.7 (0.5, 1.0)
	Women	1.2 (0.2, 9.8)	1.4 (0.2, 10.7)	1.3 (0.2, 10.5)	0.7 (0.4, 1.1)	0.6 (0.4, 1.0)	0.6 (0.4, 1.0)
	Men	1.9 (0.6, 5.8)	1.9 (0.6, 6.0)	2.0 (0.6, 6.1)	0.9 (0.4, 1.9)	0.9 (0.4, 1.9)	0.9 (0.4, 1.9)
Nursing/Health care	Total	1.5 (0.8, 2.8)	1.7 (0.9, 3.1)	1.6 (0.9, 2.9)	1.3 (1.1, 1.5)	1.3 (1.1, 1.5)	1.2 (1.0, 1.4)
	Women	2.4 (1.1, 5.5)	2.4 (1.1, 5.3)	2.2 (1.0, 4.9)	1.2 (1.0, 1.4)	1.2 (1.0, 1.4)	1.1 (1.0, 1.3)
	Men	1.0 (0.4, 2.8)	1.0 (0.3, 2.8)	0.9 (0.3, 2.7)	1.7 (1.1, 2.6)	1.5 (1.0, 2.4)	1.5 (0.9, 2.2)
Art studies	Total	2.2 (0.7, 7.4)	1.7 (0.5, 5.6)	1.6 (0.5, 5.4)	1.0 (0.6, 1.5)	1.0 (0.7, 1.5)	1.0 (0.6, 1.5)
	Women	2.5 (0.3, 19.9)	2.6 (0.3, 20.6)	2.5 (0.3, 19.4)	0.9 (0.5, 1.6)	0.8 (0.5, 1.5)	0.8 (0.5, 1.4)
	Men	1.3 (0.3, 5.7)	1.3 (0.3, 5.7)	1.3 (0.3, 5.6)	1.5 (0.8, 2.9)	1.4 (0.7, 2.8)	1.4 (0.7,2.7)
Subgroup programs							
Medical students	Total	2.2 (0.6, 7.2)	1.7 (0.5, 5.8)	1.7 (0.5, 5.9)	0.7 (0.4, 1.1)	0.7 (0.4, 1.1)	0.7 (0.4, 1.1)
	Women	NA	NA	NA	0.7 (0.4, 1.2)	0.6 (0.3, 1.1)	0.5 (0.3, 1.0)
	Men	2.0 (0.6,7.3)	2.1 (0.6,7.4)	2.2 (0.6, 7.9)	1.1 (0.5, 2.5)	1.1 (0.5, 2.4)	1.1 (0.5, 2.4)
Nursing students	Total	1.6 (0.8, 3.0)	1.7 (0.9, 3.4)	1.6 (0.8, 3.1)	1.6 (1.4, 1.9)	1.5 (1.3, 1.8)	1.4 (1.2, 1.7)
	Women	2.6 (1.1, 6.1)	2.4 (1.0, 5.8)	0.9 (0.3, 3.2)	1.5 (1.2, 1.7)	1.5 (1.3, 1.8)	1.3 (1.1, 1,6)
	Men	1.0 (0.3, 3.4)	0.9 (0.3, 3.3)	0.9 (0.3, 3.2)	2.2 (1.4, 3.4)	2.0 (1.3, 3.2)	1.8 (1.1, 2.9)

^a^Adjusted for age and calendar year. In the total group also adjusted for sex; ^b^Adjusted for age, calendar year, previous hospitalization for mental disorder and previous hospitalization for self-harm. In the total group also adjusted for sex. NA = Not possible to analyze due to too few cases

In the total groups, adjusted for sex, age and period, higher point estimates were seen for students in Nursing/Health care, OR 1.7 (95% CI 0.9–3.1), Theology/Humanities, OR 1.9 (95% CI 0.8–4.5), Technology, OR 1.3 (95% CI 0.8–2.2) and Medicine/Dentistry, OR 1.9 (95% CI 0.7–5.0). Further adjustment for prior vulnerability showed similar results.

In the subcategorization of nursing students and medical students (Table 3), there was a higher suicide risk in female nursing students, OR 2.4** (**95% CI 1.0–5.8), but only before adjusting for prior vulnerability.

### Risk differences

Crude absolute suicide risk differences were small and significant only for women in Nursing/Health care and for nursing students only (Table 4).

**Table 4:** Crude risk differences in suicide for women and men in each university program category with 95% confidence intervals (CI)

University program category	Group	Suicide	Self− harm
		Risk difference (1/10,000) (95% CI)	Risk difference (1/10,000) (95% CI)
Education (reference)		Ref.	Ref.
Theology/Humanities	Women	1.6 (− 1.3, 4.4)	13.2 (1.1, 2.5)
	Men	1.0 (− 4.1, 6.1)	0.9 (− 8.9, 10.6)
Law/Social sciences	Women	0.0 (− 0.9, 0.9)	0.5 (− 4.8, 5.8)
	Men	− 0.8 (− 3.5, 1.9)	4.6 (− 1.5, 10.1)
Natural sciences	Women	2.8 (− 0.8, 6.5)	− 0.6 (− 11.4, 10.2)
	Men	NA	3.3 (− 7.9, 14.4)
Technology	Women	− 0.5 (− 1.3, 0.3)	− 11.5 (− 16.6, − 6.4)
	Men	0.6 (− 1.8, 0.3)	− 5.8 (− 10.4, − 1.2)
Agriculture/Forestry	Women	NA	− 15.5 (− 16.5, − 3.3)
	Men	0.7 (− 6.9, 5.5)	− 12.7 (− 20.0, − 5.4)
Medicine/Dentistry	Women	0.2 (− 2.0, 2.4)	− 9.6 (− 19.5, 0.2)
	Men	3.3 (− 3.9, 10.4)	− 1.8 (− 12.4, 8.7)
Nursing/Health care	Women	1.3 (0.0, 2.4)	6.7 (1.3, 12.1)
	Men	0.0 (− 3.9, 3.9)	10.9 (1.2, 20.6)
Art studies	Women	1.3 (− 3.0, 5.7)	− 3.8 (− 19.1, 11.5)
	Men	0.1 (− 5.8, 7.8)	7.7 (− 7.4, 22.8)
*Subgroup programs*			
Medical students	Women	NA	− 10.3 (− 23.0, 2.5)
	Men	3.9 (− 5.0, 12.7)	1.9 (− 11.8, 15.7)
Nursing students	Women	1.4 (0.0, 2.8)	13.6 (7.1, 20.1)
	Men	− 0.2 (− 4.7, 4.4)	18.5 (5.4, 31.7)

*NA* Not possible to calculate due to too few cases

### Self-harm after registration in different university programs

A total of 1433 students had at least one self-harm event registered within three years of entering a university program category. Women accounted for the majority of self-harm events, *n* = 1053 (73%) (Table 2).

The risk of self-harm, after adjustment for age, sex and period, was increased for the total group in Nursing/Health care, OR 1.3** (**95% CI 1.1–1.5) compared to the reference category. It was also higher for men and women separately (Table 3). The subcategorization showed that nursing students of both sexes had a higher risk of self-harm, and that the association remained after adjusting for prior vulnerability (Table 3).

A lower risk of self-harm after initial adjustment was found in Technology, in the total group, OR 0.6 (95% CI 0.5–0.7), as well as for women in the sex-stratified analysis, OR 0.6 (95% CI 0.5–0.7) (Table 3). Students in Agriculture/Forestry had a lower risk of self-harm in the total group only.

No difference in risk of self-harm was seen in the category of Medicine/Dentistry or in the subcategorization of medical students only (Table 3).

### Risk differences

The risk differences for self-harm were mostly not statistically significant. The risk was slightly higher only for students in Nursing/Health care, for nursing students only of both sexes, and for women in Theology/Humanities (Table 4).

## Discussion

In this national cohort study, we found that female students who enrolled in university programs within nursing and health care and natural sciences had a higher risk of suicide. We also found that Nursing/Health care students had a higher risk of self-harm. A subcategorization of nursing students confirmed a higher risk of suicide in female students and of self-harm in both sexes. This pattern partly reflects suicide risk differences for working professionals in the same areas [1, 2, 8, 26, 27].

### Students in nursing/health care and nursing students

Our finding that female students in Nursing/Health care had a higher risk of suicide in the first three years following registration at university is to our knowledge unprecedented and provides an important addition to the ongoing research in finding reasons for the increased suicide risk in nurses. We confirmed that the association was mainly driven by those aiming to become nurses. The increased risk was partly affected by the comparatively high prevalence of previous mental disorder and self-harm found in this category. However, the finding that adjustment for prior vulnerability did only marginally decrease the strength of the association indicates that there may exist prior vulnerability factors not measured in the present study, such as personality traits or a family history of suicide. Also, not all mental disorders or acts of self-harm lead to contacts with healthcare services. Furthermore, additional risk factors associated with the university years could contribute to the increased suicide risk, and symptoms of mental health problems and mental illnesses may first emerge in early adulthood [28].

Our results are in line with previous findings of higher suicide risk in nurses [1, 2, 8]. In similarity with our finding that the elevated suicide risk was confined to women in the Nursing/Health care category and to nursing students specifically, a U.S. study found a higher risk for female nurses but not for male nurses. A similar gender difference, with a higher risk for women only in the group of life science and health professionals, was found in a recent Swedish study of suicide in different occupations [29].

This elevated risk of self-harm found in the group of Nursing/Health care students and in nursing students warrants attention since acts of self-harm have been shown to increase the risk of future suicide [30, 31]. Since we investigated self-harm events recorded in inpatient care, it is probable that we captured the most severe cases, and that the actual self-harm prevalence is higher. Our results indicate that the onset of vulnerability for suicide in nurses exists prior to entering the labor market.

### Students in medicine/dentistry

Consistent with several studies that have shown an increased risk of suicide in physicians, the estimates for suicide in the category Medicine/Dentistry were elevated, albeit outside the conventional level of statistical significance [1, 2, 4]. Results did not change after adjusting for previous vulnerability, which could be due to vulnerability factors not covered by our strict vulnerability measure, only comprising hospitalizations, or suggest that factors belonging to the study environment of different university programs also contribute to suicide risk. Our results are in contrast with previous findings of a higher suicide risk in female physicians [2, 4, 32, 33]. This could suggest that work-related factors rather than prior vulnerability contribute to suicide risk in female physicians.

Although educational systems differ between countries, our finding of an elevated suicide risk estimate in Medicine/Dentistry is partly in line with the Japanese study that found a higher risk of suicide in medical students [22]. When we subcategorized medical students only, the estimate remained elevated for men only. These statistically non-significant findings may be related to issues of power and length of follow-up. The Japanese study found that the suicide risk was highest in medical students who repeated semesters, took leaves of absence and were in the final years of education [22]. It is possible that the suicide risk estimates in Medicine/Dentistry in the present study would have been higher with a longer follow-up period.

The category of Medicine/Dentistry also included dentistry students, veterinary students and optician students who may have contributed to the elevated suicide risk estimate. Elevated risks of suicide have previously been reported in dentists and veterinarians [2, 10, 34].

In Medicine/Dentistry as well as in the subcategorization of medical students only, there was no increased risk of self-harm which may signify that for this student category self-harm is less likely to precede suicide. A Norwegian survey study found that while lifetime suicidal thoughts were common in physicians, there was a low rate of previous suicide attempts [35].

### Students in other program categories

Despite being a broad category with diverse programs, the elevated suicide risk found in female students in Natural sciences could suggest that there is a vulnerability for suicide in women aiming for natural science-oriented professions, a group possibly included in the life science and health professionals group of the Swedish study mentioned previously where women had an elevated risk of suicide [29].

The higher suicide risk estimates found in Theology/Humanities differed from the finding of a lower risk of suicide in students of the humanities in the study from Japan [22]. This could partly have been due to the program categories of the studies including disparate programs, making comparison difficult. Our finding suggests that there may be an increased risk for suicide in persons aiming for work in fields related to the programs included in this category, such as media professions. A previous U.S. study found that women working in arts, design, entertainment, sports, and media had the highest suicide rate [26].

### Strengths

The main strengths of this study include the total population-based cohort design, the large sample size, the complete nationwide coverage of the study exposures, outcomes and covariates and the complete follow-up using valid national Swedish registers with negligible missing data [23]. In addition, concerns of underreporting of suicides, mainly lifted in US literature, is less of a problem with our Cause of Death Register [36, 37].

We have been able to adjust for previous mental disorder and any previous self-harm requiring hospitalization, which to our knowledge, is unprecedented in previous research of university program students and suicide. By doing so, we could show that a difference in risk of each outcome in those entering some programs was not attributable to severe previous vulnerability. There is, however, a possibility of influence from vulnerability factors not covered by this study, such as a family history of suicide or mental disorder, early adverse life events, other medical conditions, or mental disorders, and in particular suicidal behavior, which have not led to hospitalization.

### Limitations

Despite our national coverage, there were few cases of suicide in each program category, which requires some caution when interpreting the results. We cannot exclude the possibility that certain specific programs within the broadest categories are associated with either or both outcomes even when no statistically significant association was found for the whole group. Furthermore, we cannot exclude the presence of residual confounding.

The results in combined categories of students may not be entirely representative of specific occupations. However, our subcategorization of medical students and nursing students should be comparable with professional nurses and physicians.

Our measure of vulnerability is, as already mentioned, conservative, consisting only of inpatient care data. We acknowledge that there is thus a risk of underestimation of pre-course mental health problems and self-harm incidence. However, our intention was not to cover the previous prevalence of nonsuicidal self-injury, which is less likely to lead to hospitalization [38]. Rather, the term self-harm instead of suicide attempt was selected for this outcome to signal that we had no information on suicidal intent.

Due to the scarcity of previous research in this area, and the different settings between our study and the previous Japanese longitudinal study, more research is needed to verify our findings and determine whether they may be generalized to other areas in the world. Furthermore, even if nurses may have similar work environments and tasks globally, we may not know whether selection patterns into different future occupations are similar between Sweden and the U.S. where elevated suicide rates among nurses was reported [8].

Although we have not considered competing risks, the young age of the study populations and the low number of suicide deaths inclines us to believe that this did not pose a problem.

Not all individuals who register for a university program remain registered during the entire follow-up period. However, our primary intention was to study whether there may be a selection of individuals into different program categories regardless of educational achievement.

### Conclusion

The elevated suicide risk in nursing and health care professions is mirrored by an increased risk of suicidal behavior during university education. This may be due to a selection of more vulnerable individuals into these occupations, but also due to factors within the educational environment contributing to the onset of suicidality. Increased efforts in identifying and treating mental disorders and preventing self-harm in university students could be an important step in preventing future suicides.

## Data Availability

Data for this study are not freely available upon demand due to regulations imposed by the Swedish national register holders to protect the personal integrity of the persons whose data were used and to abide by ethical rules.

## Declarations

### Code availability

SAS and STATA syntax is available upon request.
