# Correction to: Risk of suicide and self-harm in university students entering different university programs – a national register-based cohort study in Sweden

**DOI:** 10.1007/s00127-023-02512-1

**Published:** 2023-06-05

**Authors:** Christine Takami Lageborn, Johan Bjureberg, Jie Song, Bo Runeson, Jette Möller, Rickard Ljung, Marie Dahlin

**Affiliations:** 1 Centre for Psychiatry Research, Department of Clinical Neuroscience, Karolinska Institutet and Stockholm Health Care Services, Region Stockholm, Norra Stockholms Psykiatri, Vårdvägen 1, 112 81 Stockholm, Sweden; 2 Department of Medical Epidemiology and Biostatistics, Karolinska Institutet, Stockholm, Sweden; 3 Centre for Psychiatry Research, Karolinska Institutet and Stockholm Health Care Services, Stockholm County Council, Stockholm, Sweden; 4 Department of Psychology, Stanford University, Stanford, CA USA; 5 Department of Global Public Health, Karolinska Institutet, Stockholm, Sweden; 6 Institute of Environmental Medicine, Unit of Epidemiology, Karolinska Institutet, Stockholm, Sweden

**Correction to: Social Psychiatry and Psychiatric Epidemiology** 10.1007/s0017-03-0484-2

In Table 4 of this article, the data in the row 6th headed Natural sciences were incorrected. It has been corrected. The correct Table 4 and values as follows:

**Table 4:** Crude risk differences in suicide for women and men in each university program category with 95% confidence intervals (CI)

University program category	Group	Suicide	Self-harm
		Risk difference (1/10,000) (95% CI)	Risk difference (1/10,000) (95% CI)
Education (reference)		Ref.	Ref.
Theology/Humanities	Women	1.6 (− 1.3, 4.4)	13.2 (1.1, 2.5)
	Men	1.0 (− 4.1, 6.1)	0.9 (− 8.9, 10.6)
Law/Social sciences	Women	0.0 (− 0.9, 0.9)	0.5 (− 4.8, 5.8)
	Men	− 0.8 (− 3.5, 1.9)	4.6 (− 1.5, 10.1)
Natural sciences	Women	2.8 (− 0.8, 6.5)	− 0.6 (− 11.4, 10.2)
	Men	NA	3.3 (− 7.9, 14.4)
Technology	Women	− 0.5 (− 1.3, 0.3)	− 11.5 (− 16.6, − 6.4)
	Men	0.6 (− 1.8, 0.3)	− 5.8 (− 10.4, − 1.2)
Agriculture/Forestry	Women	NA	− 15.5 (− 16.5, − 3.3)
	Men	0.7 (− 6.9, 5.5)	− 12.7 (− 20.0, − 5.4)
Medicine/Dentistry	Women	0.2 (− 2.0, 2.4)	− 9.6 (− 19.5, 0.2)
	Men	3.3 (− 3.9, 10.4)	− 1.8 (− 12.4, 8.7)
Nursing/Healthcare	Women	1.3 (0.0, 2.4)	6.7 (1.3, 12.1)
	Men	0.0 (− 3.9, 3.9)	10.9 (1.2, 20.6)
Art studies	Women	1.3 (− 3.0, 5.7)	− 3.8 (− 19.1, 11.5)
	Men	0.1 (− 5.8, 7.8)	7.7 (− 7.4, 22.8)
*Subgroup programs*			
Medical students only	Women	NA	− 10.3 (− 23.0, 2.5)
	Men	3.9 (− 5.0, 12.7)	1.9 (− 11.8, 15.7)
Nursing students only	Women	1.4 (0.0, 2.8)	13.6 (7.1, 20.1)
	Men	− 0.2 (− 4.7, 4.4)	18.5 (5.4, 31.7)

*NA *not possible to calculate due to too few cases

The original article has been corrected.

